# Measuring *E. coli* and bacteriophage DNA in cell sonicates to evaluate the CAL1 reaction as a synthetic biology standard for qPCR

**DOI:** 10.1016/j.bdq.2016.12.001

**Published:** 2016-12-29

**Authors:** Alexander Templar, Desmond M. Schofield, Darren N. Nesbeth

**Affiliations:** The Advanced Centre for Biochemical Engineering, Department of Biochemical Engineering, University College London, WC1E 6BT, UK

**Keywords:** PCR, Polymerase chain reaction, HCD, high cell density, qPCR, quantitative PCR, SF, shake flask, wcw, wet cell weight, WCB, working cell bank, LRE, linear regression of efficiency, OCF, optical calibration factor, Pcr, Synthetic biology, Standard curve, Standardisation, Linear regression, Efficiency

## Abstract

We measured the impact of the presence of total *Escherichia coli* (*E. coli*) cellular material on the performance of the Linear Regression of Efficiency (LRE) method of absolute quantitative PCR (LRE qPCR), which features the putatively universal CAL1 calibration reaction, which we propose as a synthetic biology standard. We firstly used a qPCR reaction in which a sequence present in the lone genomic BirA locus is amplified. Amplification efficiency for this reaction, a key metric for many quantitative qPCR methods, was inhibited by cellular material from bioreactor cultivation to a greater extent than material from shake flask cultivation. We then compared LRE qPCR to the Standard Curve method of absolute qPCR (SC qPCR). LRE qPCR method matched the performance of the SC qPCR when used to measure 417–4.17 × 10^7^ copies of the BirA target sequence present in a shake flask-derived cell sonicates sample, and for 97–9.7 × 10^5^ copies in the equivalent bioreactor-derived sample. A plasmid-encoded T7 bacteriophage sequence was next used to compare the methods. In the presence of cell sonicates from samples of up to OD_600_ = 160, LRE qPCR outperformed SC qPCR in the range of 1.54 × 10^8^–1.54 × 10^10^ copies of the T7 target sequence and matched SC qPCR over 1.54 × 10^4^–1.54 × 10^7^ copies. These data suggest the CAL1 standard, combined with the LRE qPCR method, represents an attractive choice as a synthetic biology qPCR standard that performs well even when unpurified industrial samples are used as the source of template material.

## Highlights

• We establish the effect of *E. coli* cellular material on sensitivity of qPCR for detection and quantitation of a lone genomic sequence.
• We demonstrate that LRE qPCR matches performance of the conventional Standard Curve qPCR method with respect to absolute quantitation of a genomic *E. coli* sequence.
• We characterise the effect of *E. coli* cellular material on performance of qPCR for detection and quantitation of a bacteriophage DNA sequence.

## Graphical abstract

**Figure:**
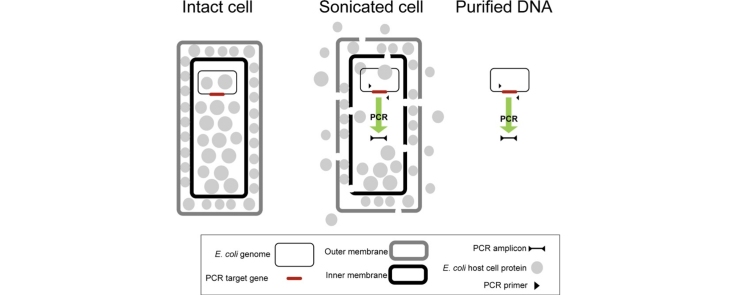

## 1 Introduction

### 1.1 LRE qPCR and the CAL1 reaction as a synthetic biology standard for qPCR

Perhaps the most widely known use of real time PCR, also known as quantitative PCR (qPCR), is as a tool to measure the relative abundance of a given messenger RNA (mRNA) transcript. In this ‘relative qPCR’ approach reverse transcriptase (RT) is used to convert a population of mRNA molecules to single stranded complimentary DNA (cDNA) molecules. A bespoke primer pair is then used to amplify cDNA corresponding to an mRNA whose abundance is well characterised. Further primer pairs are used to amplify cDNA corresponding to mRNA molecules whose abundance is unknown. Ideally, primers for qPCR will be designed in accordance with the ‘Minimum Information for Publication of Quantitative Real-Time PCR Experiments’ or ‘MIQE’ guidelines proposed by Bustin et al. [5].

The amount of PCR product (amplicon) present during each reaction cycle can be determined by measuring in real time [9] the light emission from a fluorescent reporter dye that binds to double-stranded DNA (dsDNA). The kinetics of the appearance of fluorescence over time can then be used to infer the amount of template cDNA that was initially present [20]. A key metric for this procedure is the number of cycles required for fluorescence to exceed a set threshold. This cycle number is known as the quantification cycle (Cq). The less cycles required for fluorescence to reach Cq, the more template was present in the starting material. For relative qPCR the principle data gathered is the fold-difference in abundance of the well-characterised, reference mRNA in comparison to the mRNA of unknown abundance.

**Fig. 1:**
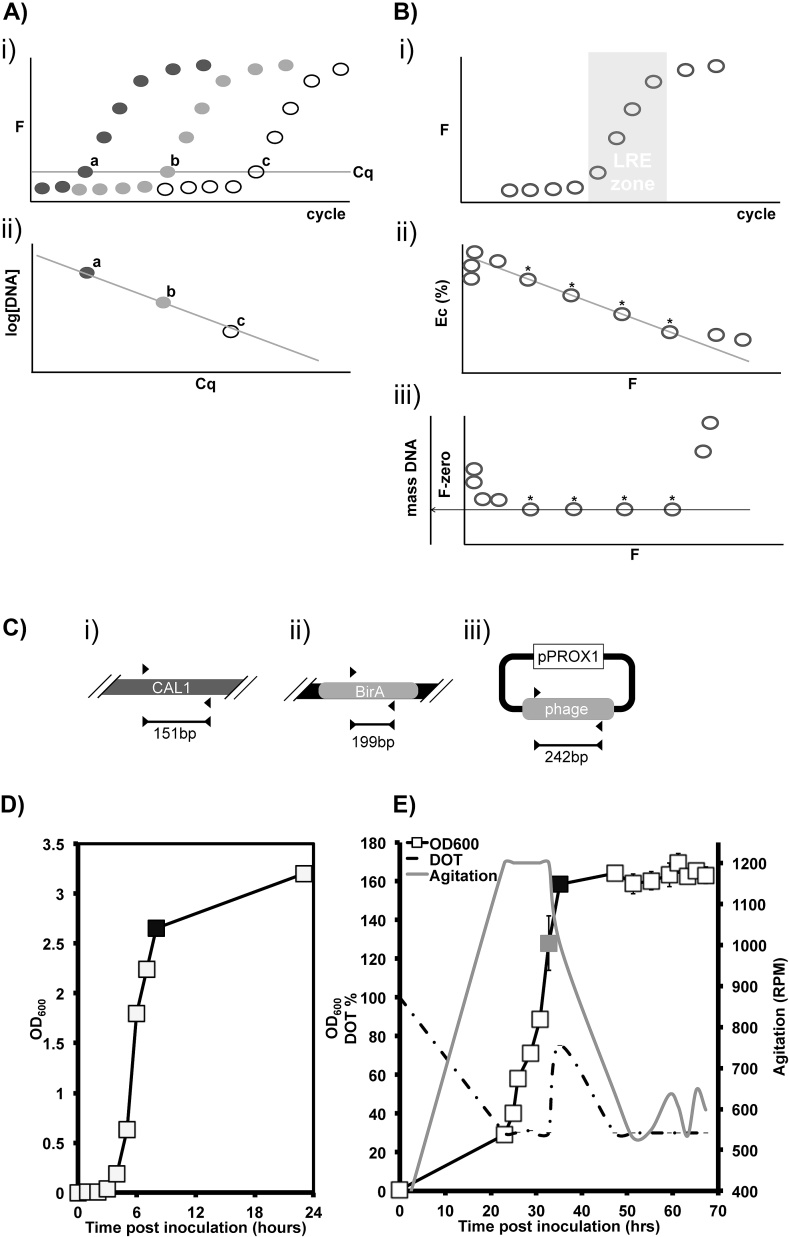
PCR approaches and cell cultivation. A) i) Illustration of the fluorescence data profile for a conventional qPCR experiment. Serial dilutions of template are made and real time appearance of fluorescence plotted as a function of cycle number. Fluorescent data points for three dilutions of template are indicated by black, grey and white (with black border) data points to convey increasing template dilution. Typically four or more are used in actual qPCR experiments. The point at which each fluorescence signal reaches the Cq is logged (cycles a, b and c). ii) Cq number is proportional to the log of the DNA concentration in purified target sequence samples of known concentration. This data set provides a standard curve for calibration of Cq data gathered from samples of unknown target sequence concentration. B) i) For LRE qPCR these is no inherent requirement to perform a template dilution series or set a Cq threshold. Instead the flourescence data set is analysed as a classic Boltzmann sigmoid function such that a sub-set of data points is identified at the midpoint of the amplification profile, known as the LRE zone (in grey). ii) Fluorescence data points within the LRE zone (indicated by asterisks) have a linear relationship to a value defined by Rutledge and Stewart [28] as cycle efficiency (Ec). iii) Rutledge and Stewart [28] also relate these LRE zone data points to the original mass of template present, expressed as a fluorescence value (F-zero). F-zero can be converted to template DNA mass using an OCF, for which CAL1 has been identified as the superior candidate. C) Primers (black triangles), detailed in Table 1, were used to amplify i) the designated CAL1 locus with the lambda bacteriophage genome, ii) target DNA within the BirA locus of the *E. coli* genome and iii) a bacteriophage sequence present in the plasmid pPROX1 as a proxy for pathogen detection. Expected amplicon size (bp) is indicated under the bar at the bottom of each panel. D) A 40 mL culture of *E. coli* W3110 production strain grown in LB was used to inoculate 360 mL defined media in a 5L shake-flask. An uninduced sample was taken at the start of stationary phase growth in shake flasks (black filled square) for PCR experiments. E) 10% of shake flask culture was used to inoculate 3.6 L defined media in a New Brunswick 7L bioreactor. In bioreactor cultivation, IPTG was added to induce transgene expression at 34 h post-inoculation (grey filled square) and a sample taken 2 h post-induction (black filled square) for PCR experiments. Agitiation, grey line, and dissolved oxygen tension (DOT), dashed line, were plotted alongside cell growth. Both cell growth data sets are representative of n = 3 experiments.

While relative qPCR has been immensely valuable in helping researchers gain fundamental biological insights, it is arguably less well suited to the aim of synthetic biology, which is to render biological systems more amenable to rigorous engineering methods. Fortunately for synthetic biologists it is possible to derive absolute measurements using qPCR [23]. Serially diluted standards of known concentration produce a linear relationship between the Cq value and the logarithm of the initial amount of total template DNA (Fig. 1A). Comparison with such a standard curve (SC) allows exact inference of the number of template molecules that were originally present in a given sample.

The reliability of absolute, standard curve qPCR (SC qPCR) depends on the efficiency of template amplification for the target and the standard curve, both of which must be evaluated. Most often, the SC is comprised of the same primer pair and template that are used for the experimental samples in which the amount of template is not known. Such a standard curve is ideally performed alongside every experiment, increasing the time taken to perform the assay, and is unique to each target so is inherently unsuited to global standardisation. SC qPCR also assumes constant amplification efficiency across all template dilutions, but in reality this is seldom observed [26].

Rutledge and Stewart [28] proposed a universal standard for absolute qPCR as an element of their Linear Regression of Efficiency method of absolute qPCR (LRE qPCR). LRE qPCR does not require a target-specific standard curve, assume constant amplification efficiency across all template dilutions or involve determination of Cq values (Fig. 1B). In LRE qPCR, original template quantity is inferred instead by applying a Boltzmann sigmoidal statistical framework to raw fluorescence data in the central area of an amplification profile [38]. Other linear regression approaches to analysis of qPCR fluorescence data have been developed, a selection of which are discussed by [24]. However, in the case of LRE qPCR, Rutledge and Stewart [28] also took pains to demonstrate that the fluorescence intensity of SYBR Green I [37] generated during real-time PCR is not impacted by either amplicon size or guanine:cytosine (GC) base pair (bp) content. Spandidos et al. [32] also reported this property of SYBR Green I. This means that absolute fluorescence units (FU) can be correlated with base pair production (FU/bp).

Using absolute units for measurement makes standardisation possible by replacing target-by-target standard curves with a single, well-defined universal standard. The CAL1 reaction, consisting of a pair of 27 bp primers specific for amplification of a 151 bp amplicon from lambda bacteriophage genomic DNA (Fig. 1C), was shown by Rutledge and Stewart [28] to exhibit amplification and fluorescence performance with a high degree of reliability over multiple experiments in a 4 month period. As such the CAL1 reaction is a strong candidate for adoption as a universal qPCR standard.

A single universal quantitative standard for qPCR could better enable monitoring of inter-run performance and benchmarking for comparison of sample preparation procedures, reaction methods, instrumentation, and data processing. Absolute measurements could enable a move away from relative measures of gene expression units [39], enabling cell status to play a greater role in quantifying performance of synthetic gene networks.

### 1.2 Factors that impact PCR accuracy in industrial settings

The sequence specificity and accuracy [17] of PCR have made it an effective assay for detection of bioprocess contamination, most commonly for mycoplasma in mammalian host chassis cultivation [1], but also of bacteriophage in *E. coli* cultivation [36]. Typically a sample is removed from cultivation and tested using end point PCR (e-pPCR) with primers specific for a target sequence present in the contaminant organism genome. Gel electrophoresis is then used to score the absence or presence of an amplicon band as an indicator of the presence or absence of the contaminant organism within the sample [12].

Factors such as gene expression [13], plasmid copy number [16] and the dosage of defined loci within a genome [22], [11] can all impact industrial performance of engineered cells. All these factors can potentially be monitored using qPCR, given a sufficiently rapid procedure. Bacteriophage can compromise virtually any industrial process involving bacteria [33], even those with comprehensively refactored genomes [14] and as such rapid and accurate detection of bacteriophage within industrial conditions is highly desirable.

Sample processing [15] represents a significant proportion of the time taken to perform most qPCR protocols and is widely believed to be necessary for removal of constituents that may inhibit the reaction and its accuracy. Sample processing for PCR typically involves removal of all non-DNA macromolecules from a given sample by physicochemical means, either by a standard protocol or with commercial kits.

The trade-off of sample preparation for PCR is that the duration of the procedures can restrict application to off-line, retrospective analyses. Furthermore, some commercial sample preparation kits may introduce error through loss of target material [18], co-purification of inhibitors [30] or introduction of contaminant DNA [29]. Shortening or foregoing sample preparation in a manner that preserves the accuracy of PCR-based assays could significantly reduce assay time-scales. Combining this with recent developments in ‘ultra-rapid’ thermal cycling speeds (Xxpress™ PCR, London, UK) [7] and electrophoresis tech- nology (FlashGel™, Lonza, Basel, Switzerland) [8] could make PCR a realistic analytical procedure for at-line bioprocess monitoring.

### 1.3 Aims of this study

In this study we compare performance of LRE qPCR and SC qPCR methods using i) material from shake flask and high cell density bioreactor cultivation and ii) measuring a genomic locus and a plasmid-encoded bacteriophage sequence as a targets. We will also discuss the suitability of LRE qPCR as a synthetic biology standard for qPCR.

## 2 Materials and methods

All reagents were of molecular biology grade unless otherwise stated. All stocks, solutions and reagents were prepared with molecular biology grade water (Millipore, Billerica, USA), confirmed DNA and ribonuclease free by the supplier. Oligonucleotides were synthesised by Eurofins MWG Operon (Acton, UK, www.eurofinsdna.com).

### 2.1 Cultivation of *E. coli*

An *E. coli* W3110 production strain that harbours the 3010 bp plasmid pTTOD-A33 which encodes a recombinant Fab’ fragment [19] inducible by addition of isopropyl β-d-1-thiogalactopyranoside (IPTG) was grown in Luria Bertani (LB) medium. 40 mL of this LB inoculum was used to inoculate 360 mL chemically defined media [2] in a 5L shake-flask to OD_600_ = 2.5 (Fig. 1D), typical of the end-point of seed train cultivation used to provide inoculum for growth in bioreactors. 10% of this shake flask material was used to inoculate 3.6 L defined media in a New Brunswick 7L bioreactor. “A fed batch protocol was used as previously described by Balasundaram et al. [2] until OD_600_ = 130 and a spike in dissolved oxygen tension (DOT) were reached, indicating complete consumption of the starting batch of glycerol”. From this point on fed-batch mode was applied along with IPTG addition to induce Fab' fragment expression (Fig. 1E). An experimental sample was taken two hours post-induction, at OD_600_ = 160, during early idiophase growth when bacteriophage contamination can be highly costly.

### 2.2 Total nucleic acid purification

**Fig. 2:**
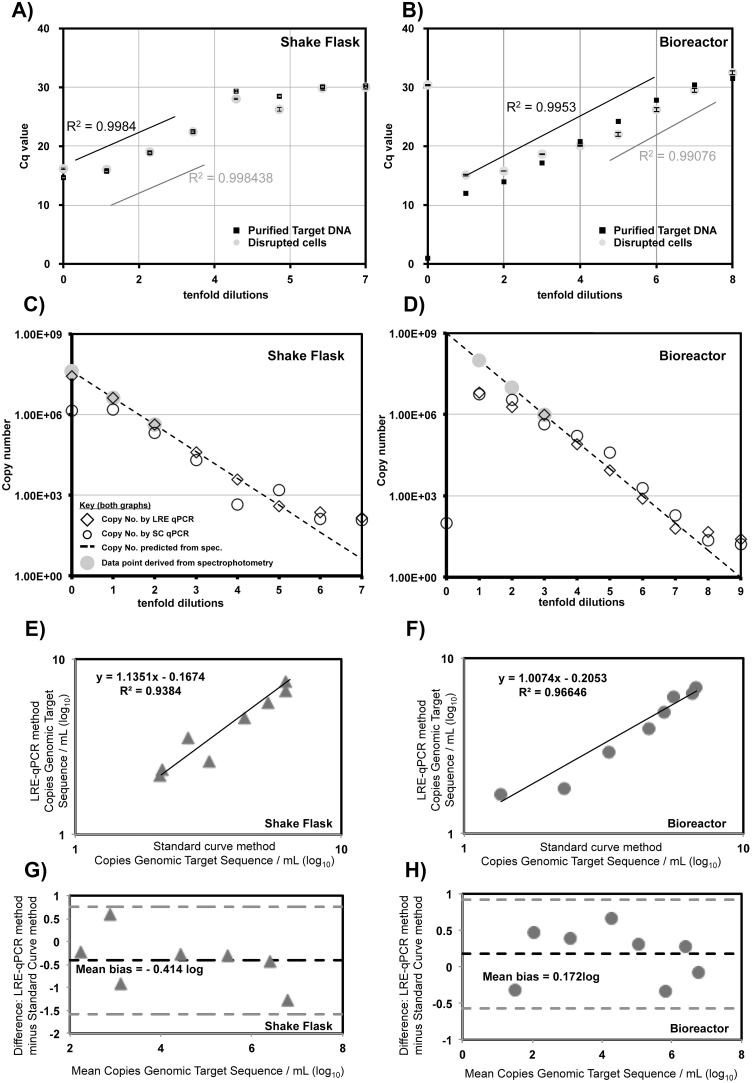
Influence of disrupted *E. coli* cells on LRE and SC qPCR. Real time PCR was performed using template material either from bioreactor sonicates (grey triangles and lines) or purified DNA (black squares and lines) from OD_600_ = 2.5 shake flask (A) and OD_600_ = 160 bioreactor (B) cultivation. Spectrophotometric estimation indicated that a 5 μL undiluted, purified DNA sample derived from shake flask material contained 112 ng total DNA (2.17 × 10^7^ copies BirA target) and a 5 μL sonicated cell sample contained 215 ng total (4.17 × 10^7^ copies BirA target). For bioreactor material, 3.9 μg (7.57 × 10^8^ copies BirA target) and 5 μg (9.7 × 10^8^ copies BirA target) respectively of DNA was present in a 5 μL purified gDNA and 5 μL cell sonicate samples respectively. Lines indicate iterative least-square fitting of a linear function to data points until a set was identified with 100 ± 10% efficiency, at a confidence level of R^2^ > 0.99. Error bars show standard error across n = 3 analytical repeats. C) Copy number estimation in a shake flask derived sample was determined by three spectrophotometric measurements (grey circles) which were linearly extrapolated (dashed lines) and plotted alongside copy numbers determined by SC qPCR (circles) and LRE qPCR (rhomboids) methods. D) Copy number estimation in a bioreactor derived sample was determined by three spectrophotometric measurements (grey circles), linear extrapolation of that date (dashed lines) and plotted alongside SC qPCR (circles) and LRE qPCR (rhomboids) data. E) XY plot (grey triangles) comparison of SC qPCR and LRE qPCR shake flask data sets from graph A. F) XY plot (grey circles) comparison of SC qPCR and LRE qPCR bioreactor data sets from graph B. G) Bland-Altman analysis of graph C plots the difference between the X and Y data points (grey triangles) and the overall average difference between the X and Y data (dark dashed lines). 1.96 x the standard deviation (+/−) of this bias (grey dashed lines) is also plotted to indicate the upper and lower limits of statistical significance [4]. H) Equivalent Bland-Altman analysis of bioreactor data (grey circles) from graph D.

**Fig. 3:**
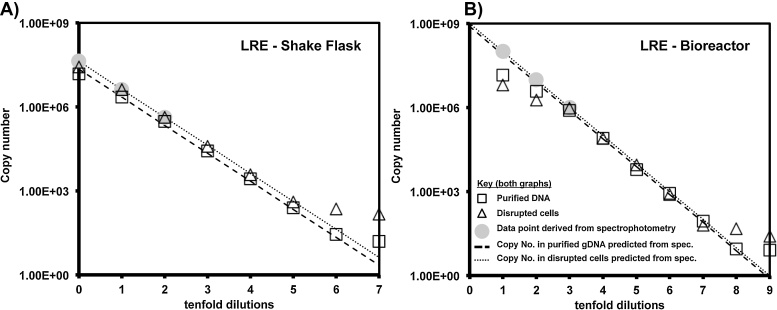
Influence of disrupted *E. coli* cells on LRE qPCR quantification of an *E. coli* genomic target sequence. The LRE method was applied to real time PCR fluorescence data gathered using dilutions of shake flask (graph A) and bioreactor material (graph B) and pure gDNA extracted from these materials. Grey data points indicate spectrophotometric data and dashed lines extrapolate these data to predict copy number at lower dilutions. The undiluted samples from shake flask and bioreactor cultivation contain the same cell and DNA content as detailed in Fig. 2.

DNA was purified by the method below from the shake flask and bioreactor samples to determine typical DNA measurements by spectrophotometry. After this initial scoping study samples ranging in volume from 400 μL–8 mL were used to provide the DNA concentration in the undiluted template reactions indicated in Figs. 2 and 3. Samples were centrifuged at 10,000 RPM for 3 min, re-suspended in 400 μL lysis buffer (2% Triton X100, 1% SDS, 100 mM NaCl, 10 mM Tris-HCl, 1 mM EDTA) and freeze-thawed twice by incubating at −80 °C for 3 min and 95 °C for 1 min. Total nucleic acid was purified using a standard phenol/ethanol extraction procedure [35] and the purified DNA was resuspended in 400 μL 10 mM Tris (pH 7.5) for both shake flask and bioreactor-derived samples. Six aliquots of purified DNA were made and stored at −20 °C. A given aliquot was thawed once for experimentation and any unused portion of the aliquot discarded. The proxy plasmid pPROX1 was purified by standard plasmid DNA purification using a Key Prep mini prep kit (Anachem, Luton, UK).

### 2.3 Cell disruption

Shake flask and bioreactor samples were sonicated using the procedure below to determine typical DNA estimations by spectrophotometry and densitometry. After this initial scoping study, the volume of sample, from 400 μL–4 mL, required to provide the final estimated DNA concentrations indicated in Fig. 2B, C and Fig. 3 was centrifuged at 10,000 RPM for three minutes and re-suspended in 400 μL dH_2_O. A Soniprep 150 sonicator (MSE, London, UK) was used to subject samples to three cycles of the following treatment: 10 s pulses of 100% amplitude sonication followed by 10 s rest, for a total duration of 60 s, 30 s of which is the total period during which cells were subjected to sonication. For PCR experiments with bacteriophage target, bioreactor sonicate was diluted with dH_2_O to an equivalent OD_600_ of 5 or 50, using the original of OD_600_ of 160 to inform the volumetric calculations.

### 2.4 PCR primers and proxy plasmid design

The CAL1 primer pair (Fig. 1C, Table 1) directs amplification of a specific 151 bp region of the Lambda bacteriophage genome [6]. Due to the amplification performance of this 151 bp region, Rutledge and Stewart [28] designated the CAL1 reaction an Optical Calibration Factor (OCF) suitable for use as a global standard reaction for LRE qPCR. We use the CAL1 standard and apply the LRE qPCR derivation of fluorescent data using a Java program developed and maintained by Rutledge [27]. Primer sequences (Table 1) were designed in accordance with MIQE guidelines [5] and screened *in silico* for specificity and potential for self-annealing using the National Center for Biotechnology Information (NCBI) ‘primer blast’ tool (http://www.ncbi.nlm.nih.gov/tools/primer-blast/accessed 22.03.15) and the ‘PCR primer stats’ tool (http://www.bioinformatics.org/sms2/pcr_primer_stats.html accessed 22.03.15) respectively.

A 300 bp proprietary bacteriophage target sequence is present within a pUC57 backbone in the 3010 bp plasmid pPROX1 (Fig. 1C) and 21 bp primers were used for amplicon production. The use of a proxy sequence in this way enables investigation of detection of many pathogen types without the need to risk infection of other cultivation experiments being performed in the same facility.

After sample processing, pPROX1 was added at known concentration and the ability of PCR methods to detect or quantify the bacteriophage sequence was tested. The BirA locus (Gene ID: 12934397) of *E. coli* W3110 strain was chosen as a single copy genomic target locus (Fig. 1C, Table 1). CAL1 primers, amplification target and PCR conditions set out by Rutledge and Stewart [28] were used to calibrate LRE qPCR experiments. Agarose gel electrophoresis showed all three reactions produced only amplicon of expected size.

### 2.5 DNA mass estimation by spectrophotometry and densitometry

DNA mass in nucleic acid purified samples and sonicated material was estimated by spectrophotometry, using a Nanodrop 1000 spectrophotometer (Thermo Scientific, Waltham, MA, USA). Sonicated samples were also analysed on a 1% agarose gel stained with ethidium bromide. Stained DNA was visualised using a GelDoc 2000 (BioRad, Hercules, CA, USA) and Quantity One software version 4.6.8 (BioRad, Hercules, CA, USA). ImageJ software (version 1.46r, National Institutes of Health, Bethesda, USA) was used to select a region of the gel image, either a lane or a band, containing a known mass of DNA and a brightness value captured. A selected region of the same size and shape was then used to capture the brightness level in a region of unknown DNA concentration on the same gel. Background noise was subtracted using ImageJ ‘subtract background’ command, details of which can be found at this support website − http://imagej.net/Rolling_Ball_Background_Subtraction.

### 2.6 Quantitative PCR assembly and data capture

Reactions were carried out in a total volume of 20 μL, with each reaction containing 10 μL of 2 x SsoAdvanced SYBR Green Supermix (BioRad, Hercules, CA, USA), 5 μL of material containing template DNA and 1 μL of each primer to give a final concentration of 500 nM. Reactions were performed in a CFX Connect Real-time PCR Detection System (Bio-Rad, Hercules, CA, USA) with a cover heated to 105 °C. Each reaction was run at a total of 40 cycles, with the same cycling conditions as above. Cq values were generated using Bio-Rad CFX manager 3.0 (BioRad, Hercules, CA, USA) and exported to Microsoft Excel 2010 for analysis. For BirA quantification experiments white Hard-Shell^®^ Low-Profile Thin-Wall 96-Well Skirted PCR plates (Bio-Rad, #HSP9601) were used and for bacteriophage quantification clear Multiplate™ Low-Profile 96-Well Unskirted PCR plates (Bio-Rad, #MLL9601) were used (Supplementary Fig. 1).

### 2.7 Determination of amplicon production efficiency

Efficiency was calculated with the standard curve method, as set out by Rutledge and Côté [26], from iterative least-square fitting of a linear function to various data points until a set of points within the desired limit of acceptance (R^2^ threshold of 0.99) was identified. Linear regression was then applied to calculate efficiency (E), with the equation:

$$E=10^{\left(\frac{-1}{slope}\right)}$$

### 2.8 Copies of target DNA determined by ‘Standard Curve’ qPCR

The standard curve generated as described above was used to estimate copies of target in samples contaminated by cell debris. Cq values of contaminated samples were plotted along the standard curve and converted into copy number using the equation:

$$targetcopynumber=10^{\left(\frac{Cq-b}{m}\right)}$$

Where b is the y-intercept and m is the slope of the standard curve.

### 2.9 Copies of target DNA determined by LRE qPCR

LRE qPCR, as described by Rutledge and Côté [26], was applied to estimate copy numbers. Lambda DNA for CAL1 calibration was purchased from New England BioLabs (Ipswich, MA, USA), product code N3011S. LRE analyser v. 0.97 [27] was used according to developer’s instructions. Purified lambda genome DNA samples of known DNA mass were used to calibrate the program and provide information on copy number in samples contaminated with cell debris.

## 3 Results

### 3.1 qPCR amplification efficiency for a genomic target in cellular material

Efficiency of amplicon production, which can be defined as the slope of Cq values when plotted as a function of reaction cycle, is key to many of the numerous statistical approaches to qPCR data analysis. Typically, efficiency within the range of 100 ± 10%, with a confidence value (r^2^) of at least 0.99, is set as the limit for accurate quantitation. Many methods assume equal amplification efficiency across all reactions in a dilution series [26].

The genomic target sequence we used is present in the *BirA* gene, which is known to be present as a single copy in the *E. coli* genome. Assuming an *E. coli* W3110 genome size of 4,646,332 bp plus 20 replicons/cell of the 6480 bp plasmid pTTOD-A33, host genomic DNA (gDNA) should constitute 97.2% of total host cell DNA (total size of DNA per cell of 4,775,932 bp). We used these assumptions to convert DNA concentration levels, derived from spectrophotometry, into target sequence copy numbers.

A sample containing 217 ng of purified DNA from shake flask material (Fig. 2A) exhibited acceptable amplification efficiency across only 3 reactions (2.17 × 10^6^–2.17 × 10^4^ copies of the BirA target − estimated by spectrophotometry) from a dilution series of 7 reactions. By contrast, 3.9 μg DNA purified from bioreactor material (Fig. 2B) showed acceptable amplification efficiency across 5 reactions (9.7 × 10^6^ to 970 copies of the BirA target − estimated by spectrophotometry) from a total of 8 tenfold dilutions. The reasons for this difference are unclear but taken together these profiles serve to illustrate further that equal amplification efficiency across a dilution series cannot be safely assumed.

For the shake flasks sample, when sonicated cells were used as template the number of acceptably efficient reactions was unchanged from the purified DNA template, at 3 (Fig. 2A). The presence of sonicated cells from the bioreactor sample reduced the number of acceptably efficient reactions to 3, down from 5 with purified DNA (Fig. 2B).

### 3.2 LRE and SC qPCR quantification of a genomic target in cellular material

Standardisation is a non-trivial goal in biology [21] and a range of statistical approaches exist for method comparison [10]. Here we assess the equivalence of SC qPCR and LRE qPCR by discussion of raw data, comparison with spectrophotometric data and direct, head-to-head statistical analysis.

Shokere et al. [31] used spectrophotometry to assess the accuracy of qPCR with purified DNA as template. Unexpectedly, we observed that spectrophotometry could be used to measure DNA concentration in the presence of disrupted cells from samples of up to OD_600_ = 16 (Fig. 2D), even though we anticipated the presence of such cell debris would distort the absorbance spectra. An R value (ratio of absorbance at 260 nm/280 nm) of 1.8 is ideal for accurate spectrophotometry. R values for sonicates of material from OD_600_ = 2.5 − OD_600_ = 16 cultures were typically close to 1.3, indicating the presence of high levels of protein, and also possibly RNA, contributed significantly to the absorption at 260 nm. DNA concentrations as low as 2 ng/μL were consistently measurable by spectrophotometry of sonicated cells. The low R value meant that we considered all spectrophotometric measurements in cell sonicates strictly as crude estimations only, with their primary function to assist comparison of LRE and SC qPCR methods.

Three spectrophotometric estimations were plotted (Fig. 2C and D, grey circles) and extrapolated to provide a common benchmark for comparison of LRE qPCR and SC qPCR. For quantitation of target DNA within disrupted cell samples from shake flask growth, LRE qCPR data points matched more closely the trend of the spectrophotometric data than did SC qPCR for the undiluted sample, containing 4.17 × 10^7^ copies of the BirA targert, and across 5 further tenfold dilutions to 417 copies (Fig. 2C). For high cell density bioreactor material LRE qCPR also outperformed SC qPCR for measurement of 9.7 × 10^5^ copies and across 4 tenfold dilutions to 97 copies (Fig. 2D). A caveat for bioreactor analysis is that both LRE qPCR and SC qPCR were unable to quantitate target DNA in undiluted samples and diverged significantly from the spectrophotometric estimations for the first two tenfold dilutions.

Method comparison by XY plot [4] will result in a slope of 1.00 in the case of zero bias between methods. When compared to SC qPCR using an XY plot, LRE qPCR showed a small degree of proportional bias (slope of 1.1351) for quantitation of target in disrupted cells from shake flask cultivation (Fig. 2E). The Y intercept of 0.16674 also suggested modest systematic bias. A Bland-Altman [3] plot of these data (Fig. 2G) indicated LRE qPCR had a slightly negative bias of SC qPCR data but that the methods were equivalent due to the fact that the mean bias range included zero difference [4]. XY plot comparison of LRE qPCR and SC qPCR analysis of disrupted cells from bioreactor cultivation (Fig. 2F) showed less proportional bias (slope of 1.0074) than for shake flask material but greater systemic bias (Y intercept of 0.2053). Bland-Altman plot (Fig. 2H) again indicated the methods were equivalent, as the mean bias range spanned the zero difference level [4].

### 3.3 LRE qPCR quantification of a genomic target in absence and presence of cellular material

SC qPCR is predicated on the use of a standard curve comprised of the same primers and target as those used in experimental reactions. Because purified DNA was used as standard curve for the SC qPCR experiment in Fig. 2C and D it cannot meaningfully be used to evaluate the accuracy of SC qPCR for purified DNA template. No such restriction applies to LRE qPCR and as such we plotted LRE qPCR data gathered using naked DNA and disrupted cell suspension as template alongside data points generated by spectrophotometry (Fig. 3).

For disrupted cell samples derived from shake flask cultivation (Fig. 3A), there was close agreement between LRE qPCR and spectrophotometric estimation, for undiluted material (4.17 × 10^7^ copies) and over five dilutions (to 417 copies). The copy numbers indicated by LRE qPCR plateaued for dilutions 6 and 7 (41.7 copies and 4.17 copies), suggesting either a false positive or that a certain level of DNA remains permanently associated with cellular material.

For samples derived from bioreactor cultivation the target numbers indicated by LRE qPCR were depressed for the undiluted sample of OD_600_ = 160 (5 μg, 9.71 × 10^8^ copies of BirA target) and the next 2 tenfold dilutions (Fig. 3B). LRE qPCR closely matched spectrophotometric estimation of copy number over 3–8 tenfold dilutions for purified target DNA (7.57 × 10^5^ to 7.57 copies) and 3–7 tenfold dilutions (9.7 × 10^5^ to 97 copies) in the presence of disrupted cells (Fig. 3B).

### 3.4 qPCR amplification efficiency for a bacteriophage target in cellular material

**Fig. 4:**
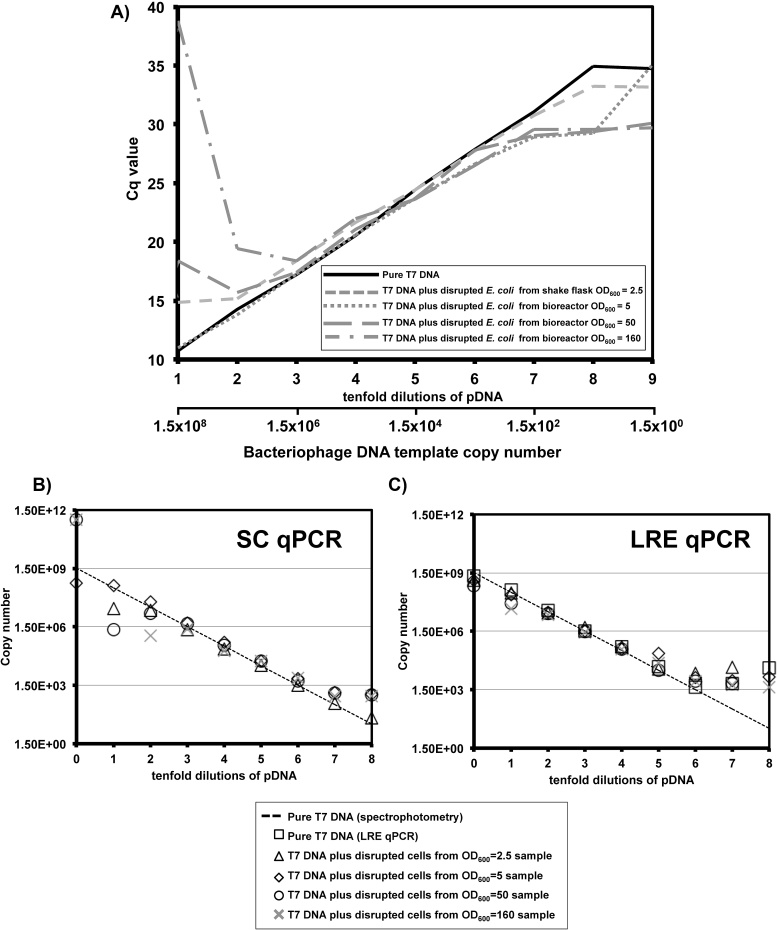
Influence of disrupted *E. coli* on qPCR analysis of a bacteriophage target sequence. 5 ng (1.54 × 10^9^ copies) of purified pPROX1 plasmid (encoding a bacteriophage target sequence) was used as template (in 1 μL) and a series of tenfold dilutions made, each with 4 μL of dH_2_O or cell sonicate added prior to amplification. A) Cq values derived from real time fluorescence data were plotted as a function of tenfold dilutions of the plasmid. Copy numbers derived from B) SC qPCR and **C**) LRE qPCR methods were also plotted alongside copy number estimates extrapolated from spectrophotometric measurements of purified plasmid DNA (dashed lines).

For quantification of a genomic target (Fig. 2, Fig. 3) cell sonicates were the only source of template and were diluted tenfold for each reaction. So while the amount of template decreased, the ratio of target to cellular material was maintained in each dilution. For industrial scale cultivation of *E. coli*, quantification of a contaminant organism such as bacteriophage is ideally effective at very low concentrations of contaminant. Consequently, for qPCR experiments with bacteriophage sequences we performed serial dilutions of the plasmid first, and then added the same amount of cell sonicate material to every dilution, decreasing the relative amount of target versus cells for each dilution (Fig. 4).

We performed real time PCR with 5 ng of the naked pPROX1 plasmid (1.54 × 10^9^ copies), encoding bacteriophage DNA sequence, as template and plotted Cq values as a function of tenfold dilutions (Fig. 4A) to assess amplification efficiency. For naked template DNA, efficiency within the range of 100 ± 10%, with a confidence value r^2^ ≥ 0.99, was observed from the sample that had undergone one tenfold dilution (1.54 × 10^9^ copies, 5 ng) to the eighth tenfold dilution (154 copies, 500ag). The lowest level of naked template DNA, 15.4 copies (50ag), in the ninth tenfold dilution, gave the same Cq value as 154 copies.

### 3.5 LRE and SC qPCR quantification of bacteriophage target in cellular material

We next used LRE qPCR and SC qPCR to derive absolute bacteriophage DNA copy numbers in the presence of cellular material from shake flask and bioreactor cultivation. For comparison, absolute copy numbers calculated by the two different methods were plotted alongside copy numbers derived from spectrophotometry of naked plasmid DNA.

Copy numbers calculated using the LRE qPCR method were in agreement with spectrophotometric data in the range of 1.54 × 10^8^–1.54 × 10^6^ copies (500pg–5 pg pDNA) of target, despite the presence of bioreactor material of OD_600_ up to 160 in the undiluted sample (Fig. 4C). LRE qPCR was less effective for quantification of 1.5 × 10^4^ or less copies of bacteriophage target sequence (Fig. 4C).

SC qPCR data diverged from spectrophotometric estimations at the high, 1.54 × 10^9^ copies (5 ng), and low, 150 copies (500ag), extremes of bacteriophage DNA concentration that were tested (Fig. 4B). SC qPCR had less range than LRE qPCR overall, but did show impressive accuracy extending to a copy number as low as 150 (Fig. 4B).

## 4 Discussion

### 4.1 Quantifying the need for sample preparation

A PCR-based assay that requires little or no sample preparation has the potential to help accelerate assay turnaround sufficiently to be used for at-line monitoring of bioindustrial processes [37] and the pressing challenges of scaling up synthetic biology solutions.

Efficiency of amplification underlies many statistical approaches to analysis of real time PCR data and in some approaches is assumed to be equal across all reactions. Amplification efficiency for a genomic target was not equal across all reactions, even for naked DNA (Fig. 2). Relative to amplification efficiency for naked DNA, disrupted cells from bioreactor cultivation had an inhibitory effect (Fig. 2B) that was not observed for shake flask material (Fig. 2A). This implies that amplification efficiency should be monitored for qPCR methods that assume equal amplification efficiency across all reactions and that qPCR sample preparation is more critical for bioreactor-derived cellular material.

Amplification efficiency for 1.54 × 10^7^ to 1540 copies (3–7 tenfold dilutions) of a bacteriophage target sequence was not influenced by the presence of cells sonicates from cultures of up to OD_600_ = 160. Cell sonicates from OD_600_ = 2.5 culture permitted adequate amplification efficiency for as few as 154 copies (8 tenfold dilutions) of the bacteriophage target sequence (Fig. 4A). This indicates that DNA purification is unnecessary for quantification of T7 bacteriophage DNA by qPCR and that instead a brief sonication step will enable accurate quantitation of as few as 1500 copies of target in a given sample.

### 4.2 Comparing SC qPCR and LRE qPCR

SC qPCR and LRE qPCR were directly compared with respect to their agreement with spectrophotometric data for estimation of genomic target DNA concentration in samples from shake flask (Fig. 2C) and bioreactor (Fig. 2D) cultivation. LRE qPCR data matched extrapolated spectrophotometric data for 6 out 8 data points for shake flake samples (Fig. 2C) and 5 out 10 data points for bioreactor samples (Fig. 2D). This compares to at most 3 matching data points for SC qPCR. Further statistical analysis (Fig. 2E–H) suggested the methods could be regarded as equivalent.

For quantification of a bacteriophage target, LRE qPCR was more robust to the presence of cellular material from up to OD_600_ = 160 bioreactor cultivation (Fig. 4C), while SC qPCR had a more restricted range but was able to match the copy numbers predicted by spectrophotometry down to 154 copies in the presence of cellular material of OD_600_ = 2.5 from shake flask cultivation (Fig. 4B). We suggest LRE qPCR is more suited to a future at-line bioprocess monitoring application due to its robustness to high levels of cellular material.

### 4.3 Influence of sample preparation on LRE qPCR

Comparing LRE qPCR and spectrophotometric estimations of pure DNA and cell sonicate samples from shake flask (Fig. 3A) and bioreactor (Fig. 3B) cultivation allowed us to assess the need for sample processing with this method.

For quantitation of a genomic target, shake flask samples of OD_600_ = 2.5 still allowed quantitation of 4.17 × 10^7^ copies of target (215 ng DNA) in agreement with a spectrophotometric estimate (Fig. 3A). For bioreactor samples, dilution to OD_600_ = 0.16 was necessary for quantitation of 9.71 × 10^5^ copies of target (5 ng DNA) in agreement with a spectrophotometric estimate (Fig. 3B). By contrast, quantitation in agreement with a spectrophotometric estimate was still possible in the presence of up to OD_600_ = 160 cellular material for 1.54 × 10^5^ copies (0.5 ng pDNA) of a bacteriophage target sequence (Fig. 4C). These data suggest a simple processing step, with no DNA purification, followed by 2–3 tenfold dilutions, may be sufficient to render an industrial process stream sample amenable to qPCR analysis by the LRE qPCR method.

For detection of a genomic target the presence of cellular material derived from bioreactor cultivation compromised quantitation by LRE qPCR more than when a comparable amount of shake flask material was present (Fig. 3). Accurate quantitation of target in 215 ng DNA in the presence of OD_600_ = 2.5 shake flask culture (Fig. 3A) was not matched for 50 ng in presence of OD_600_ = 1.6 bioreactor material (Fig. 3B, second tenfold dilution). The reason for the reduced LRE qPCR accuracy with bioreactor material is unknown, but the observation is consistent with the data from Fig. 2B that bioreactor material had a greater effect on amplification efficiency than shake flask material. This putative inhibitory effect of bioreactor material was not observed for detection of a bacteriophage target sequence present in a plasmid (Fig. 4C). An interaction between cellular material and DNA may be influenced by the provenance of the cells (shake flask or bioreactor cultivation) and the nature of the target (plasmid or genomic DNA). However, any confounding effects arising from such factors can be readily surmounted by sample dilution.

## 5 Conclusions

The accuracy profile of LRE qPCR matched that of SC qPCR by the measures performed here using industrially relevant samples. In light of these observations, and previous validation of the properties of LRE qPCR [25], we invite the synthetic biology community to use test CAL1 standard and LRE qPCR procedure for absolute qPCR. Accumulation of data and experience could lead to the establishment of CAL1 as a useful synthetic biology standard.

## Figures and Tables

**Table 1:** Oligonucleotide primers for PCR.

*Target*	Primers	Sequence
*E. coli* BirA gene	BirA Fwd	ATCCACCCCTGATTAACGAC
	Rev BirA	CGGAAGTATTACGCAAGCTG
Lambda OCF region	Cal 1 Fwd	AGACGAATGCCAGGTCATCTGAAACAG
	Rev CAL1	CTTTTGCTCTGCGATGCTGATACCG
300 bp bacteriophage sequence	Forward	21mer
	Reverse	21mer
