# Supplementary material for: Measuring E. coli and bacteriophage DNA in cell sonicates to evaluate the CAL1 reaction as a synthetic biology standard for qPCR
Source: Biomol Detect Quantif. 2016 Dec 29;11:21–30. doi: 10.1016/j.bdq.2016.12.001 (PMC5348119; doi:10.1016/j.bdq.2016.12.001)
Supplement: Supplementary file 1 [file mmc1.docx]

**Supplementary Figures.**

**Figure 1. Raw flourescence data. A**) Data in Figure 3A shake flask and 3B (bioreactor) of the report were derived from the raw fluorescence data provided here. Data set numbering refers to number of tenfold dilutions, 0-7 for shake flask material and 0-12 for bioreactor material. Colour coding distinguishes each of 3 analytical repeats of each dilution. **B**) Data in Figure 6.3, 6.4 and 6.5 of the report were derived from the raw fluorescence data provided here. Data set numbering refers to number of tenfold dilutions: 0-7 for shake flask (SF) material and 0-12 for bioreactor (BR) and numbers refer to OD_600_ level (OD_600_= 2.5, 5, 50 or 160). Colour coding distinguishes each of 3 analytical repeats of each dilution (n=2 for BR 160 material). Higher relative fluourscence unit (RFU) levels in panel B) data sets were achieved by switching to from white to clear 96 well plates (see Materials and Methods in report). This does not affect quantitation because, as set out by Rutledge and Stewert (2008), the rate of loss of cycle efficiency plus the maximum efficiency are the principle outputs of these data, which that are then converted to DNA mass using the CAL1 reaction as OCF.
