# Supplementary material for: Measuring E. coli and bacteriophage DNA in cell sonicates to evaluate the CAL1 reaction as a synthetic biology standard for qPCR
Source: Biomol Detect Quantif. 2016 Dec 29;11:21–30. doi: 10.1016/j.bdq.2016.12.001 (PMC5348119; doi:10.1016/j.bdq.2016.12.001)
Supplement: Supplementary file 2 [file mmc2.pdf]

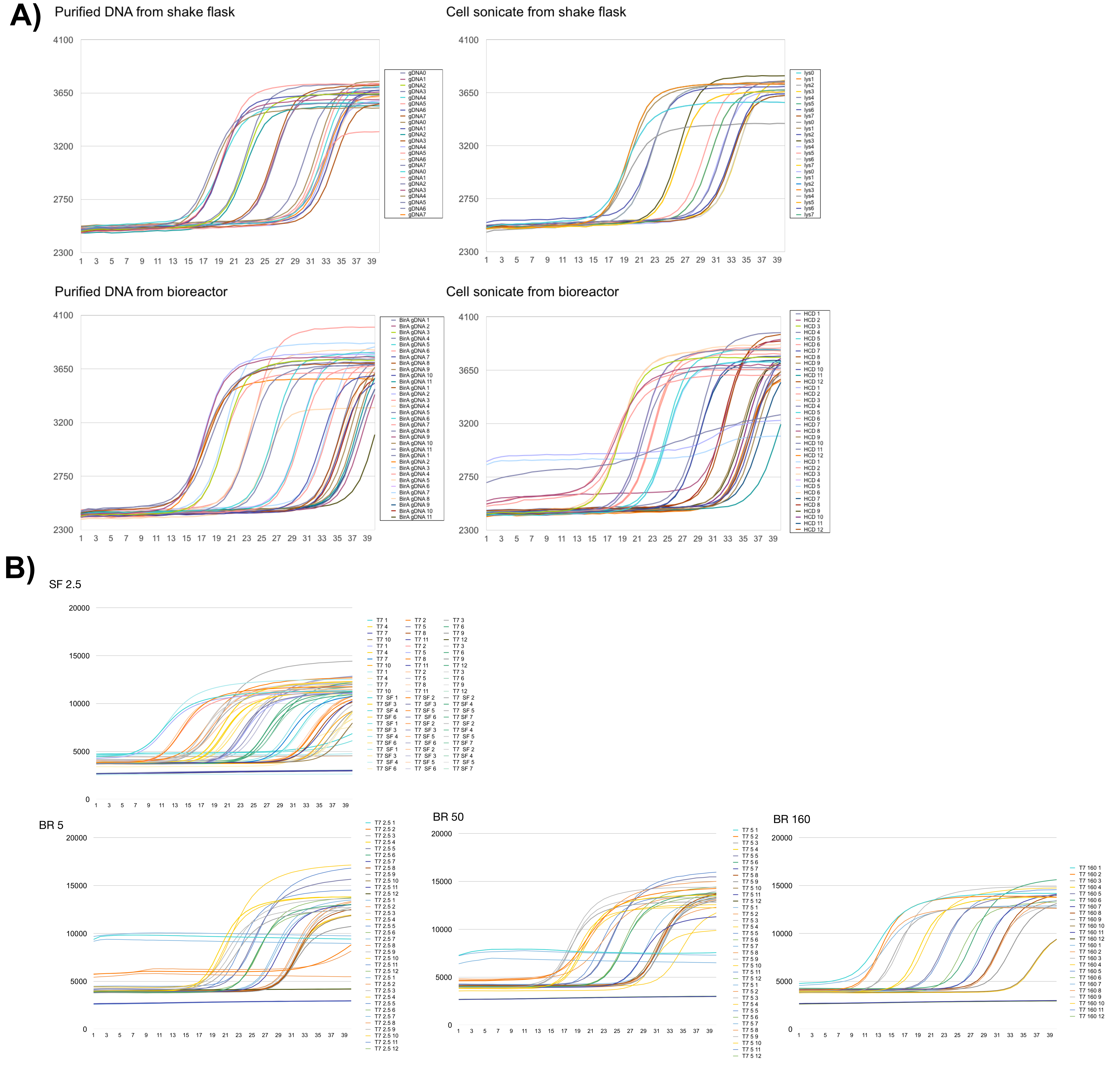
A)
Purified DNA from shake flask
4100
3650
3200
2750
2300
1 3 5 7 9 11 13 15 17 19 21 23 25 27 29 31 33 35 37 39
gDNA0 gDNA1 gDNA2 gDNA3 gDNA4 gDNA5 gDNA6 gDNA7
gDNA0 gDNA1 gDNA2 gDNA3 gDNA4 gDNA5 gDNA6 gDNA7
gDNA0 gDNA1 gDNA2 gDNA3 gDNA4 gDNA5 gDNA6 gDNA7
Cell sonicate from shake flask
4100
3650
3200
2750
2300
1 3 5 7 9 11 13 15 17 19 21 23 25 27 29 31 33 35 37 39
lys0 lys1 lys2 lys3 lys4 lys5 lys6 lys7
lys0 lys1 lys2 lys3 lys4 lys5 lys6 lys7
lys0 lys1 lys2 lys3 lys4 lys5 lys6 lys7
Purified DNA from bioreactor
4100
3650
3200
2750
2300
1 3 5 7 9 11 13 15 17 19 21 23 25 27 29 31 33 35 37 39
BirA gDNA 1 BirA gDNA 2 BirA gDNA 3 BirA gDNA 4 BirA gDNA 5 BirA gDNA 6 BirA gDNA 7 BirA gDNA 8 BirA gDNA 9 BirA gDNA 10 BirA gDNA 11
BirA gDNA 1 BirA gDNA 2 BirA gDNA 3 BirA gDNA 4 BirA gDNA 5 BirA gDNA 6 BirA gDNA 7 BirA gDNA 8 BirA gDNA 9 BirA gDNA 10 BirA gDNA 11
BirA gDNA 1 BirA gDNA 2 BirA gDNA 3 BirA gDNA 4 BirA gDNA 5 BirA gDNA 6 BirA gDNA 7 BirA gDNA 8 BirA gDNA 9 BirA gDNA 10 BirA gDNA 11
Cell sonicate from bioreactor
4100
3650
3200
2750
2300
1 3 5 7 9 11 13 15 17 19 21 23 25 27 29 31 33 35 37 39
HCD 1 HCD 2 HCD 3 HCD 4 HCD 5 HCD 6 HCD 7 HCD 8 HCD 9 HCD 10 HCD 11 HCD 12
HCD 1 HCD 2 HCD 3 HCD 4 HCD 5 HCD 6 HCD 7 HCD 8 HCD 9 HCD 10 HCD 11 HCD 12
HCD 1 HCD 2 HCD 3 HCD 4 HCD 5 HCD 6 HCD 7 HCD 8 HCD 9 HCD 10 HCD 11 HCD 12
B)
SF 2.5
20000
15000
10000
5000
0
1 3 5 7 9 11 13 15 17 19 21 23 25 27 29 31 33 35 37 39
T7 1 T7 2 T7 3 T7 4 T7 5 T7 6 T7 7 T7 8 T7 9 T7 10 T7 11 T7 12
T7 1 T7 2 T7 3 T7 4 T7 5 T7 6 T7 7 T7 8 T7 9 T7 10 T7 11 T7 12
T7 1 T7 2 T7 3 T7 4 T7 5 T7 6 T7 7 T7 8 T7 9 T7 10 T7 11 T7 12
T7 SF 1 T7 SF 2 T7 SF 2 T7 SF 3 T7 SF 3 T7 SF 4 T7 SF 4 T7 SF 5 T7 SF 5 T7 SF 6 T7 SF 6 T7 SF 7
T7 SF 1 T7 SF 2 T7 SF 2 T7 SF 3 T7 SF 3 T7 SF 4 T7 SF 4 T7 SF 5 T7 SF 5 T7 SF 6 T7 SF 6 T7 SF 7
T7 SF 1 T7 SF 2 T7 SF 2 T7 SF 3 T7 SF 3 T7 SF 4 T7 SF 4 T7 SF 5 T7 SF 5 T7 SF 6 T7 SF 6 T7 SF 7
BR 5
20000
15000
10000
5000
0
1 3 5 7 9 11 13 15 17 19 21 23 25 27 29 31 33 35 37 39
T7 2.5 1 T7 2.5 2 T7 2.5 3 T7 2.5 4 T7 2.5 5 T7 2.5 6 T7 2.5 7 T7 2.5 8 T7 2.5 9 T7 2.5 10 T7 2.5 11 T7 2.5 12
T7 2.5 1 T7 2.5 2 T7 2.5 3 T7 2.5 4 T7 2.5 5 T7 2.5 6 T7 2.5 7 T7 2.5 8 T7 2.5 9 T7 2.5 10 T7 2.5 11 T7 2.5 12
T7 2.5 1 T7 2.5 2 T7 2.5 3 T7 2.5 4 T7 2.5 5 T7 2.5 6 T7 2.5 7 T7 2.5 8 T7 2.5 9 T7 2.5 10 T7 2.5 11 T7 2.5 12
BR 50
20000
15000
10000
5000
0
1 3 5 7 9 11 13 15 17 19 21 23 25 27 29 31 33 35 37 39
T7 5 1 T7 5 2 T7 5 3 T7 5 4 T7 5 5 T7 5 6 T7 5 7 T7 5 8 T7 5 9 T7 5 10 T7 5 11 T7 5 12
T7 5 1 T7 5 2 T7 5 3 T7 5 4 T7 5 5 T7 5 6 T7 5 7 T7 5 8 T7 5 9 T7 5 10 T7 5 11 T7 5 12
T7 5 1 T7 5 2 T7 5 3 T7 5 4 T7 5 5 T7 5 6 T7 5 7 T7 5 8 T7 5 9 T7 5 10 T7 5 11 T7 5 12
BR 160
20000
15000
10000
5000
0
1 3 5 7 9 11 13 15 17 19 21 23 25 27 29 31 33 35 37 39
T7 160 1 T7 160 2 T7 160 3 T7 160 4 T7 160 5 T7 160 6 T7 160 7 T7 160 8 T7 160 9 T7 160 10 T7 160 11 T7 160 12
T7 160 1 T7 160 2 T7 160 3 T7 160 4 T7 160 5 T7 160 6 T7 160 7 T7 160 8 T7 160 9 T7 160 10 T7 160 11 T7 160 12
